# Hemispheric Lateralization of Motor Thresholds in Relation to Stuttering

**DOI:** 10.1371/journal.pone.0076824

**Published:** 2013-10-11

**Authors:** Per A. Alm, Ragnhild Karlsson, Madeleine Sundberg, Hans W. Axelson

**Affiliations:** 1 Department of Neuroscience, Speech and Language Pathology, Uppsala University, Uppsala, Sweden; 2 Department of Neuroscience, Clinical Neurophysiology, Uppsala University, Uppsala, Sweden; University of Reading, United Kingdom

## Abstract

Stuttering is a complex speech disorder. Previous studies indicate a tendency towards elevated motor threshold for the left hemisphere, as measured using transcranial magnetic stimulation (TMS). This may reflect a monohemispheric motor system impairment. The purpose of the study was to investigate the relative side-to-side difference (asymmetry) and the absolute levels of motor threshold for the hand area, using TMS in adults who stutter (n = 15) and in controls (n = 15). In accordance with the hypothesis, the groups differed significantly regarding the relative side-to-side difference of finger motor threshold (p = 0.0026), with the stuttering group showing higher motor threshold of the left hemisphere in relation to the right. Also the absolute level of the finger motor threshold for the left hemisphere differed between the groups (p = 0.049). The obtained results, together with previous investigations, provide support for the hypothesis that stuttering tends to be related to left hemisphere motor impairment, and possibly to a dysfunctional state of bilateral speech motor control.

## Introduction

### Dysregulation of Speech Muscle Activation in Stuttering

Stuttering is a complex speech motor disorder, characterized by intermittent inability to move forward in the speech sequence. The exact nature of the speech disruptions is still a matter of debate. Many theorists have assumed that excessive muscular tension is a core feature of the disorder, which can be exemplified by the influential definition: “Stuttering is an anticipatory, apprehensive, *hypertonic* avoidance reaction” [1]. However, some studies of speech muscle activation in persons who stutter have reported a tendency toward relatively low levels of functional muscle activation during speech [2]–[4]. One possibility is that stuttering involves both insufficient activation of relevant speech muscles and excessive involuntary tension, as two sides of dysregulated speech muscle activation.

### Indications of Elevated Motor Threshold in Stuttering

Transcranial magnetic stimulation (TMS) can be used to investigate the neurophysiological basis of movement disorders through measurement of motor evoked potentials (MEPs). Stuttering shares some clinical traits with task-specific dystonia, such as task-specific disturbance of motor control involving excessive muscular activation, aggravation by emotional stress, and genetic predisposition. Neurophysiologically it has been shown that task-specific dystonia is associated with reduced intracortical inhibition, tested with paired-pulse TMS [5]. The similarites between dystonia and stuttering motivated investigation of the intracortical excitability of the left hemisphere of persons who stutter [5]. However, the stuttering group showed normal results, which did not support the analogy with dystonia. An unexpected finding of the study was that the stuttering group showed significantly higher motor threshold (MT) for elicitation of finger MEPs from the left hemisphere, compared with the controls, see Sommer et al. (2003) in Table 1. As judged by their data, the stuttering group showed large heterogeneity in this respect, with about 2 to 3 times higher standard deviation of the scores compared with the controls. Because stuttering mainly has been associated with excessive muscular activity the finding of elevated motor threshold was counterintuitive.

**Table 1:** Summary of published MT values of stuttering adults and control group, for left and right cerebral hemispheres.

Study	Measure	n St	left mean		right mean		left SD		right SD	
			St	C	St	C	St	C	St	C
Sommer et al. (2003) [5]	Finger RMT	16	54.5^a^	47.3			12.1	6.5		
	Finger AMT	16	42.2^a^	34.6			11.2	4.7		
Neef et al. (2011a) [6]	Finger AMT	14	52.1	46.6	50.9	46.8	12.3	7.8	9.7	8.15
Neef et al. (2011b) [7]	Tongue, exp. 1	12	48.5	46.5	44.7	49.0	9.8	7.3	8.7	8.2
	Tongue, exp. 2	8	42.8	40.8	42.6	44.4	8.4	6.7	5.9	8.8

St = Stuttering; C = control group; RMT = resting motor threshold; AMT active motor threshold; SD = standard deviation.

In two more recent studies of MT in stuttering persons [6], [7] this group difference was less clear, see Table 1. Still, some tendencies are consistent through all studies: (a) the means and the standard deviations of the MT for the left hemisphere were somewhat higher for the stuttering groups than for the control groups; and (b) for the stuttering groups the mean left MT was somewhat higher than the right MT, while the reverse was the case for the control groups. This suggests an overall tendency towards elevation of the MT for the left hemisphere compared to the right hemisphere in the stuttering group, as well as increased heterogeneity.

### Possible Cause of Elevated Motor Thresholds in the Left Hemisphere

Several studies have reported differences in white matter microstructure of the left frontal lobe of stuttering groups, as measured by diffusion tensor imaging (DTI). Reduced values of fractional anisotropy (FA) in DTI may indicate reduced myelination or some other anomaly. The most common result in DTI-studies of stuttering appears to be reduced FA in the white matter underlying the face area of the left primary motor cortex or premotor cortex [8]–[10]. It is of interest that the motor threshold, as measured by TMS in healthy adults, has been reported to show an inverse linear relation to the FA value of the white matter underlying the primary motor cortex, with a correlation of up to *r* = 0.60 for the left hemisphere [11]. This points towards a possible link between elevation of the left hemisphere motor threshold and impairment of white matter microstructure in the left motor system in persons who stutter.

Using TMS interference it has recently been reported that right-handed persons who stutter appear to show a right-shift in the cerebral control of hand movements [6]. It was shown that stuttering adults tend to use the right dorsal premotor cortex for the timing of left hand movements, while the control subjects tended to use the corresponding left hemisphere region for this task [6]. This is in line with the other indications of left hemisphere motor impairment in persons who stutter, with subtle differences also in the motor control of the hands [12], [13].

### The Present Study

The normal inter-individual variation of MT is large, but the difference between the left and the right hemispheres is typically much smaller [14], [15]. In this way side-to-side comparison of MT within individuals or groups can be a useful method for investigation of monohemispheric disorders [14], [15]. The published data summarized above suggests that measurement of MT in stuttering persons may reveal left hemisphere anomalies in some persons who stutter. The purpose of the present study was to investigate the hemispheric side-to-side difference (asymmetry) of finger MT in persons who stutter in comparison with matched controls, and the absolute levels of finger MT for both hemispheres. Measurements of TMS-induced finger motor responses (the abductor digiti minimi muscle) were chosen to enable comparison with the previous conducted study [5], reporting elevated left hemisphere motor threshold for this muscle in stuttering adults. In addition, preliminary attempts to record from tongue or lips revealed technical problems to separate TMS artifacts from the obtained motor responses, which is not a problem for more distal muscles.

## Materials and Methods

### Ethics Statement

The study was approved by the Regional Ethical Review Board in Uppsala, Sweden, www.epn.se/en, dnr 2010/208. Information about the study was provided and written informed consent was collected prior to participation. Data regarding the measured motor thresholds is available upon request.

### Participants

Individual biographical data of the participants are presented in Table 2. The participants consisted of adults aged 20 to 52 years, with 15 persons stuttering since childhood (one female, mean age 30.0 years, *SD = *10.6) and 15 controls matched for gender, age, and handedness (mean age 29.5 years, *SD = *11.0), without known relatives with persistent stuttering. The mean severity score according to the Stuttering Severity Instrument-3, SSI-3 [16], was 23.4 (*SD = *11.9), ranging from 4 (“very mild”) to 43 (“very severe”). Handedness was determined using item 1, 2, 3, 6, and 7 from the Edinburgh Handedness Inventory [17]. The mean lateralization quotient for handedness [17] was 82 (*SD* = 53) for the stuttering group and 83 (*SD* = 52) for the controls. The exclusion criteria were medication which may affect the motor threshold, metal implants, known brain damage, previous neurosurgery, stroke, epilepsy, and other neurological disorders.

**Table 2:** Participants, biographical data.

Id	Group	Gender	Age	Handednessscores	Stuttering severity score,SSI-3	Stuttering severitylabel
1	St	m	43	100	15	Very mild
2	St	m	20	33	32	Severe
3	St	m	35	100	18	Mild
4	St	m	35	100	43	Very severe
5	St	m	25	100	37	Very severe
6	St	m	21	100	18	Mild
7	St	m	21	−100	25	Moderate
8	St	f	22	100	7	Very mild
9	St	m	21	100	missing	missing
10	St	m	20	100	38	Very severe
11	St	m	44	100	31	Moderate
12	St	m	25	100	11	Very mild
13	St	m	47	100	23	Mild
14	St	m	47	100	25	Moderate
15	St	m	24	100	4	Very mild
16	C	m	28	100		
17	C	m	22	100		
18	C	m	23	100		
19	C	f	26	−100		
20	C	m	21	75		
21	C	m	26	100		
22	C	m	25	100		
23	C	m	22	75		
24	C	m	27	100		
25	C	m	46	100		
26	C	m	33	100		
27	C	m	52	100		
28	C	m	21	100		
29	C	m	51	100		
30	C	m	20	100		

St = Stuttering; C = Control group; m = male, f = female. Stuttering severity was estimated using the Stuttering Severity Instrument-3, SSI-3 [16], Handedness score based on item 1, 2, 3, 6, and 7 from the Edinburgh Handedness Inventory [17].

### Equipment and Procedure

Resting MT was determined for a finger muscle, the abductor digiti minimi muscle. The participants sat in a reclining chair and were instructed to stay relaxed and avoid moving or talking, but to keep eyes open and to look at a computer screen showing nature sceneries. Navigated TMS was applied using a figure-of-eight coil connected to a stimulator device (Magstim 2^nd^ generation double 70 mm coil and Rapid^2^ stimulator, Magstim Company Limited, UK) [18]. The coil was positioned tangentially to the skull with the handle backwards in 45° angle to the sagittal plane. The hotspot with maximum MEP amplitude was determined by the following procedure: the likely position was marked on a tissue cap, as a 5×5 cm area centred 5 cm laterally and 1.5 cm anteriorly to of the vertex. The corners of this area were registered on a standard (single subject) 3D brain MRI using a navigation system (Visor, ANT, Netherlands). This area was used to aid the search for the “hotspot” with the highest MEP amplitudes, which were represented by color coded markers on the 3D MRI. During MT estimation at the determined hotspot, the positioning and angles of the coil were continuously monitored by the navigation system to ensure stable coil position. The MT was estimated by means of computerized adaptive parameter estimation by sequential testing (PEST) [19], [20], with the software TMS Motor Threshold Assessment Tool 2.0 [21]. For all participants the MT of the left hemisphere was estimated before the right.

### Statistics

Stimulation strengths (MT level and side-to side difference) are given in percent (0 to 100%) of maximum machine output (MO). For tests of statistical significance of group differences Mann-Whitney U-test was used, because normal distribution could not be assumed. Statistics were performed by the software Statistica 10 (StatSoft Inc.). Side-to-side difference of MT (asymmetry) was calculated as percent of the mean MT for both hemispheres.

## Results

### Side-to-Side Difference (Asymmetry) and Absolute Level of MT

**Figure 1:**
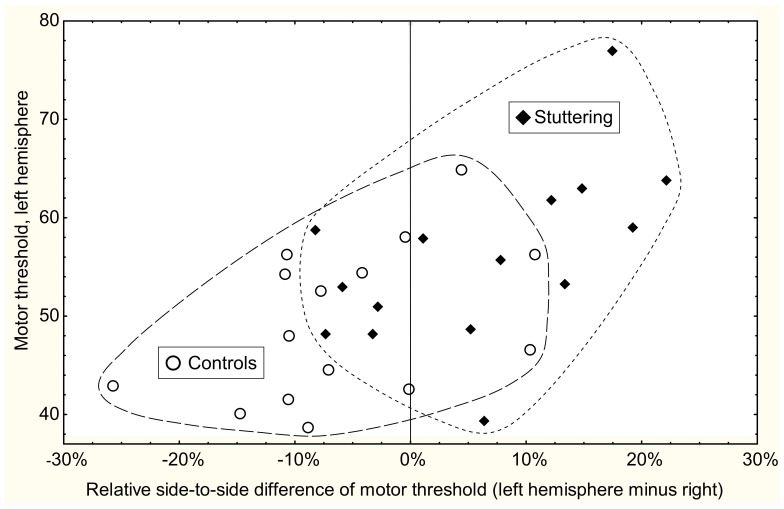
Side-to-side difference of motor threshold in relation to left hemisphere motor threshold. Scatter plot of individual motor threshold (MT) results: relative side-to-side difference of MT (left minus right, in percent of mean MT) versus MT for the left hemisphere. ♦ = stuttering, ○ = controls. The ranges for the groups are marked with dashed lines. The control group showed a tendency towards lower motor threshold for the left hemisphere, here indicated as negative values on the x-axis. The stuttering group showed an opposite tendency.

The results for MT and side-to-side differences are shown in Table 3 and Figure 1. The groups differed significantly regarding relative side-to-side difference of MT (*p = *0.0026), with the stuttering group showing higher MT of the left hemisphere in relation to the right. Also the absolute level of the MT for the left hemisphere was significantly higher in the stuttering group compared with the control group (*p = *0.049, 2-sided test), while there were no indications at all for a group difference for the right hemisphere MT. Figure 1 shows that only 3 controls (20%) had higher MT for the left hemisphere than for the right, while this was the case for 10 persons (67%) in the stuttering group. The degree of asymmetry of motor thresholds was unrelated to the severity of stuttering (*r* = 0.15, *p* = 0.62) and to handedness.

**Table 3:** Percent side-to-side difference (asymmetry) of motor thresholds (left hemisphere minus right, divided by mean MT) and absolute motor threshold levels for stuttering group and control group.

		Mean		*SD*		*p*	Effect *d*
		St	C	St	C		
%MT diff. L-R:		6.1%	−5.7%	10.2%	9.6%	0.0026*	1.20
MT	Left:	55.9	49.4	8.9	7.8	0.049*	0.77
	Right:	52.4	52.2	6.9	7.3	0.92	

Tests of significance using the Mann-Whitney U-test, and effect size calculated as Cohen’s *d.* St = Stuttering; C = control group; MT = motor threshold; SD = standard deviation.

## Discussion

### Relative Decrease of Left Hemisphere Motor Excitability in the Stuttering Group

The result of this study confirmed the hypothesis that the stuttering group tended to show relatively high MT for the left hemisphere, especially in comparison with their own right hemisphere but also in comparison with left hemispheres of matched controls. In contrast, there were no indications of differences of MT related to the right hemisphere, which showed normal levels of MT in the stuttering group. This suggests that the underlying pathology of stuttering tends to be a monohemispheric impairment related to the left speech motor system, which in some cases results in an increased MT, also for the fingers. In contrast, the fluent persons typically showed lower MT for the left hemisphere compared with the right.

This result is in line with studies of functional brain imaging of persons who stutter, which repeatedly have shown a tendency towards a rightward shift of frontal activity during speech, including the Broca’s area homologue, the ventral premotor cortex, and the mouth area of the primary motor cortex [22]. As discussed in the introduction there are several reports of impairment of white matter microstructure (low FA) underlying the left primary motor cortex or premotor cortex in persons who stutter [8]–[10]. Considering the finding of an inverse relation between the MT and the FA value of the white matter beneath the motor cortex in healthy adults [11], it appears quite possible that all of these observations of stuttering groups are causally linked: (a) impairment of the left hemisphere white matter related to the motor system, (b) a tendency towards elevation of the left hemisphere MT, and (c) a rightward shift of frontal activity during speech.

### Possible Bilateral Speech Motor Control

If some cases of stuttering are related to a relative increase of the left hemisphere MT, the next question is in what way this is causally related to the symptoms of stuttering. One possibility is that there is a direct causal link, so that an elevated MT makes it more difficult to initiate the necessary speech movements. Another possibility is that a relative increase of the left hemisphere MT is a correlate of the mechanism resulting in stuttering, but that the threshold in itself is not a factor. For example, it may be hypothesized that a relative increase of the left hemisphere MT is related to bilateral hemispheric control of speech, and that this bilateral control is the key factor in stuttering. This idea of bilaterality in stuttering persons was outlined already 1911 [23]. Speech requires a series of fast coordinated movements. Bilateral control of fast series of movements is likely to be especially prone to breakdowns, because of the relatively long time delay between the hemispheres as a result of the limited axonal conduction speed [24].

If stuttering is related to bilateral control of speech, and not to a unilateral motor control impairment per se, it would be predicted that stuttering can be resolved if unilateral control of speech can be attained. Three types of findings support this possibility. Firstly, there is a series of reports of lifelong stuttering being resolved after unilateral head injuries, surgery, or onset of multiple sclerosis [25]–[27]. The lesions may accidentally have resolved the assumed hemispheric competition. Secondly, a recent functional brain imaging study reported that persons who recovered from stuttering after puberty showed compensatory activity in the left Brodmann area 47, ventral to Broca’s area, so that speech motor control appeared to have become unilateral, with lateralization to the left [28]. Thirdly, a study of children age 9 to 12, reported children with persistent stuttering to have a lower volume of gray matter within Broca’s area (left) compared with controls. Surprisingly, this reduction was even more pronounced in a group of children with early recovery from stuttering [10]. A possible interpretation is that stuttering children with more pronounced left speech motor impairment successfully shifted lateralization of speech motor control to the right, while children with milder left side impairment remained in a state of bilateral hemispheric competition and unstable speech motor control.

In summary, a possible interpretation of available data is that many cases of stuttering are related to mild left hemisphere speech motor impairment, resulting in an unstable state of bilateral speech motor control.

### Motor Control of Hand and Finger Movements

The findings reported in this study may raise questions regarding the motor control of hand and finger movements in persons who stutter. As mentioned below there are studies indicating subtle impairments of hand motor control in persons who stutter. In finger tapping of complex sequences adults who stutter tend to need somewhat longer time to initiate the learned movement sequence [13]. This may be directly related to the motor problems shown in speech, because speech basically consists of a learned complex movement sequence.

The absolute levels of the left hemisphere motor threshold are not remarkable. This indicates that it is not the motor threshold per se that is the problem, but rather that the asymmetry is indicative of some left hemisphere motor system impairment, or anomalous motor system organization.

### Conclusions

The merged results of this and previous studies indicate that stuttering groups tend to have a rightward shift of motor system activation, with a tendency towards a relative elevation of the MT for the left hemisphere. However, stuttering groups are clearly heterogeneous in this respect. Elevation of left hemisphere MT may be related to structural anomalies, such as regional white matter impairment, underlying left motor cortex regions. The possible causal relation between this result and the symptoms of stuttering was discussed. A direct causal relation could be that elevation of the motor threshold is related to difficulties initiating necessary speech movements. An indirect model is that a relative increase of the left MT may be related to bilateral control of speech, and this bilaterality may be a central factor in stuttering.
